# Elevated Serum TNF-α/IL-1β Levels and Under-Nutrition Predict Early Mortality and Hospital Stay Burden in Pulmonary Tuberculosis

**DOI:** 10.3390/jcm14155327

**Published:** 2025-07-28

**Authors:** Ionut-Valentin Stanciu, Ariadna-Petronela Fildan, Adrian Cosmin Ilie, Cristian Oancea, Livia Stanga, Emanuela Tudorache, Felix Bratosin, Ovidiu Rosca, Iulia Bogdan, Doina-Ecaterina Tofolean, Ionela Preotesoiu, Viorica Zamfir, Elena Dantes

**Affiliations:** 1 Faculty of Medicine, “Ovidius” University of Constanta, 900470 Constanta, Romania; ionut.stanciu@365.univ-ovidius.ro (I.-V.S.); petronela.fildan@365.univ-ovidius.ro (A.-P.F.); doina.tofolean@univ-ovidius.ro (D.-E.T.); ionela.preotesoiu@365.univ-ovidius.ro (I.P.); viorica.zamfir@365.univ-ovidius.ro (V.Z.); elena.dantes@gmail.com (E.D.); 2 Doctoral School of Medicine, “Ovidius” University of Constanta, 900470 Constanta, Romania; 3 Department III Functional Sciences, Division of Public Health and Management, “Victor Babes” University of Medicine and Pharmacy Timisoara, 300041 Timisoara, Romania; ilie.adrian@umft.ro; 4 Center for Research and Innovation in Precision Medicine of Respiratory Diseases, “Victor Babes” University of Medicine and Pharmacy Timisoara, Eftimie Murgu Square 2, 300041 Timisoara, Romania; oancea@umft.ro; 5 Discipline of Microbiology, Faculty of Medicine, “Victor Babes” University of Medicine and Pharmacy Timisoara, Eftimie Murgu Square 2, 300041 Timisoara, Romania; 6 Methodological and Infectious Diseases Research Center, Department of Infectious Diseases, “Victor Babes” University of Medicine and Pharmacy Timisoara, Eftimie Murgu Square 2, 300041 Timisoara, Romania; felix.bratosin@umft.ro (F.B.); ovidiu.rosca@umft.ro (O.R.); iulia-georgiana.bogdan@umft.ro (I.B.)

**Keywords:** tuberculosis, cytokines, TNF-α, IL-1β, nutritional status, length of stay, case–control study, mortality, inflammatory markers, host directed therapy

## Abstract

**Background/Objectives:** Romania remains a tuberculosis (TB) hotspot in the European Union, yet host-derived factors of poor outcomes are poorly characterised. We quantified circulating pro-inflammatory cytokines and examined their interplay with behavioural risk factors, the nutritional status, and the clinical course in adults hospitalised with pulmonary TB. We analysed 80 adults with microbiologically confirmed pulmonary TB and 40 respiratory symptom controls; four TB patients (5%) died during hospitalisation, all within 10 days of admission. **Methods:** A retrospective analytical case–control study was conducted at the Constanța regional TB referral centre (October 2020—October 2023). Patients with smear- or culture-confirmed TB were frequency-matched by sex, 10-year age band, and BMI class to culture-negative respiratory controls at a 2:1 ratio. The patients’ serum interferon-γ (IFN-γ), interleukin-1α (IL-1α), interleukin-1β (IL-1β), and tumour-necrosis-factor-α (TNF-α) were quantified within 24 h of admission; the neutrophil/lymphocyte ratio (NLR) was extracted from full blood counts. Independent predictors of in-hospital mortality were identified by multivariable logistic regression; factors associated with the length of stay (LOS) were modelled with quasi-Poisson regression. **Results:** The median TNF-α (24.1 pg mL^−1^ vs. 16.2 pg mL^−1^; *p* = 0.009) and IL-1β (5.34 pg mL^−1^ vs. 3.67 pg mL^−1^; *p* = 0.008) were significantly higher in the TB cases than in controls. TNF-α was strongly correlated with IL-1β (ρ = 0.80; *p* < 0.001), while NLR showed weak concordance with multiplex cytokine patterns. Among the patients with TB, four early deaths (5%) exhibited a tripling of TNF-α (71.4 pg mL^−1^) and a doubling of NLR (7.8) compared with the survivors. Each 10 pg mL^−1^ rise in TNF-α independently increased the odds of in-hospital death by 1.8-fold (95% CI 1.1–3.0; *p* = 0.02). The LOS (median 29 days) was unrelated to the smoking, alcohol, or comorbidity load, but varied across BMI strata: underweight, 27 days; normal weight, 30 days; overweight, 23 days (Kruskal–Wallis *p* = 0.03). In a multivariable analysis, under-nutrition (BMI < 18.5 kg m^−2^) prolonged the LOS by 19% (IRR 1.19; 95% CI 1.05–1.34; *p* = 0.004) independently of the disease severity. **Conclusions:** A hyper-TNF-α/IL-1β systemic signature correlates with early mortality in Romanian pulmonary TB, while under-nutrition is the dominant modifiable determinant of prolonged hospitalisation. Admission algorithms that pair rapid TNF-α testing with systematic nutritional assessment could enable targeted host-directed therapy trials and optimise bed utilisation in high-burden settings.

## 1. Introduction

Tuberculosis (TB) remains the second-leading single infectious cause of death, claiming an estimated 1.30 million lives in 2023 despite decades of control efforts [1]. Romania registers one of the highest notification rates in the European Union (EU), with 48 cases per 100,000 inhabitants in 2024, compared with the EU-wide mean of ≈12 per 100,000 [2,3]. The Global Burden of Disease (GBD) 2021 analysis further projects that, if current trajectories persist, Eastern Europe will contribute disproportionately to global TB morbidity through 2035 [4].

To accelerate progress in this regard, the World Health Organization (WHO) launched the End TB Strategy in 2015, targeting a 90% mortality reduction and 80% incidence reduction by 2030 [5]. Progress in the reduction of TB is threatened by lifestyle-associated risk factors—alcohol and tobacco together explained one in five TB deaths worldwide in 2019 [6]—and by an expanding pool of multidrug-resistant TB (MDR-TB) cases. In Bucharest, MDR-TB was recently shown to erode household income, amplify psychological distress, and degrade the overall quality of life in a prospective cohort [7]. Even patients with drug-susceptible disease report persistent health-related quality-of-life deficits in Romanian surveys that use patient-reported outcome measures [8], which underscores the need for host-directed and socio-economic interventions beyond bacteriological cure.

The disease progression and sequelae of TB reflect a delicate immunological balance. Excessive tumour-necrosis-factor-α (TNF-α), interleukin-1β, and interferon-γ (IFN-γ) contribute to granuloma maintenance, yet may precipitate cachexia and cavitation when dysregulated. Recent mechanistic studies link an altered micronutrient availability (low circulating Fe/Se with high IL-6/IL-10) to impaired mycobacterial control [9], while TB-antigen-stimulated multiplex signatures (e.g., elevated CXCL10/IFN-γ ratios) independently predict unfavourable outcomes [10]. Peripheral blood markers of T-cell exhaustion (PD-1, CD244) have likewise emerged as correlates of delayed sputum conversion [11], and CXCL9/CXCL10 monitoring in cerebrospinal fluid refines the prognostication for tuberculous meningitis [12].

Systemic inflammation is further modulated by the host genetics and metabolic milieu. Circulating miR-206, for example, correlates with high TNF-α/IFN-γ levels and independently predicts drug resistance in Chinese pulmonary TB cohorts [13]. Classical risk factors such as under-nutrition [14] and diabetes mellitus [15] remain highly prevalent in Eastern Europe and synergise with tobacco- and alcohol-related immune suppression [6]. Disentangling these pathways is critical for tailoring adjunctive therapies and preventive counselling.

The length of hospital stay (LOS) synthesises biological severity with health-system performance. A 13-year Sicilian audit identified advanced age, >2 comorbidities, and MDR status as strong LOS and mortality risk factors [16]. Contemporary machine-learning models now predict prolonged postoperative LOS in spinal TB with >85% accuracy [17] and flag patients at risk for loss-to-follow-up during drug therapy [18], offering opportunities for early triage and resource optimization.

Host-directed therapy (HDT) research is expanding: metformin augments macrophage viability and restrains the growth of intracellular Mycobacterium tuberculosis while dampening excessive cytokine release [19], and metabolomic profiling after BCG vaccination has revealed plasma lipid signatures that predict trained-immunity amplitudes [20]. 

This investigation is, to our knowledge, the only Romanian study that simultaneously models TNF-α, IL-1β, and nutritional status against hospital stay. IL-1α (an early inflammasome product) and IFN γ (a late Th1 cytokine) were deliberately retained as ‘negative biological controls’ to demonstrate that not all pro-inflammatory mediators carry equal prognostic weight once pulmonary disease is clinically overt. Against this backdrop, the present retrospective case–control analysis of 120 adults managed in an Eastern Romanian tertiary centre aims to do the following: (i) delineate demographic and behavioural risk clusters, (ii) compare systemic cytokine profiles between TB cases and controls, and (iii) examine the inter-relations among inflammation, the LOS, and early mortality. The findings aim to inform personalised HDT strategies and context-specific quality-improvement initiatives.

## 2. Materials and Methods

### 2.1. Study Design and Setting

We implemented a retrospective, analytical, case–control study at a tertiary-level facility accredited as the regional referral centre for tuberculosis and complex pulmonary disorders (Level IIIB), collectively serving ≈ 2.0 million inhabitants. All diagnostic and therapeutic procedures during the study interval adhered to the Romanian National TB Control Programme (NTP) guidelines and to European Union Standards for Tuberculosis Care [21].

Ethical approval was obtained from the Ethics Committee of the University “Ovidius” Constanta, including approval for retrospective data collection and prospective analysis of consecutive patients with TB (approval number 15,134 from 5 October 2022). This study conforms to STROBE reporting recommendations for observational studies. The IRB works under Helsinki guidelines. 

### 2.2. Patient Selection and Group Allocation

All consecutive adult admissions (≥18 years) during a three-year period (between 1 October 2020 and October) were screened. Case inclusion criteria were as follows: (i) smear-positive Ziehl–Neelsen or culture-confirmed (MGIT 960) pulmonary TB; (ii) no anti-TB therapy within the previous 12 months; (iii) baseline serum available for cytokine analysis within 24 h of admission; and (iv) complete demographic and clinical data recorded in the HIS. Exclusions were isolated extrapulmonary TB, known HIV infection or immunosuppressive therapy (≥10 mg prednisolone-equivalent for >1 month, biologics), pregnancy, or transfer from another facility after >48 h of hospitalisation.

Symptomatic, culture negative respiratory patients were preferred to healthy volunteers because they undergo identical radiography, fasting blood sampling, and ward routines, which thereby minimised performance bias when comparing cytokine concentrations.

For each eligible TB case, the triage module was queried daily for adults hospitalised within a 7-day window for suspected pulmonary TB who ultimately met all control criteria: (i) respiratory symptoms ≥ 2 weeks and an abnormal chest radiograph suggestive of TB at presentation; (ii) ≥2 negative Ziehl–Neelsen smears, negative Xpert Ultra, and negative MGIT 960 culture on induced sputum or broncho-alveolar lavage; (iii) a definitive, non-tuberculous diagnosis (e.g., community-acquired pneumonia, COPD exacerbation) established by discharge; (iv) no prior TB treatment in lifetime; and (v) baseline serum obtained before administration of systemic corticosteroids or ≥24 h of antibiotics. Immunocompromised states, pregnancy, and active fungal/viral pneumonitis were exclusionary.

Controls were frequency-matched to cases by sex, 10-year age band, and BMI class (underweight, normal, overweight) at a 2:1 ratio (80 cases: 40 controls). When multiple candidates satisfied matching criteria, a random number generator selected the control. All controls underwent interferon-γ release assay (QuantiFERON-TB Gold Plus) to document latent TB infection status, which enabled stratified analyses. Post-hoc power analysis indicated that a sample of 80 cases and 40 controls provides 92% power (α = 0.05, two-tailed) to detect a 5 pg mL^−1^ difference in serum TNF-α (SD 12 pg mL^−1^) using Welch’s *t*-test. Extrapulmonary TB was excluded through targeted ultrasound and, when indicated, contrast-enhanced CT of abdomen, neck, and brain; all cerebrospinal fluid GeneXpert tests were negative. A single chart abstractor, blinded to case control status, verified all electronic records to minimise detection bias.

### 2.3. Data Collection and Variable Definitions

Demographic, socioeconomic, clinical, radiological, and laboratory variables were extracted. Variables with >10% missing data were omitted from multivariable modelling. We included the following categorical variables: (1) Smoking: current daily ≥ 1 cigarette. (2) Alcohol: occasional (≤7 units week^−1^) vs. chronic (>7 units week^−1^ for ≥6 months). (3) BMI: underweight (<18.5 kg m^−2^), normal (18.5–24.9), overweight (25.0–29.9). (4) Comorbidities: cardiovascular, metabolic, pulmonary, other (renal, hepatic). (5) Clinical severity: modified Bandim TB score (0–20). Primary outcomes were as follows: (i) all-cause in-hospital mortality, and (ii) length of stay (LOS) in days. Secondary outcomes included serum cytokine concentrations (IFN-γ, IL-1α, IL-1β, TNF-α) and neutrophil/ lymphocyte ratio (NLR).

### 2.4. Laboratory Procedures

Venous blood (10 mL) was drawn between 07:00 and 09:00 h after overnight fasting and centrifuged (1500× *g* × 10 min, 4 °C) within 30 min, after which serum aliquots were stored at −80 °C until batch analysis. Cytokines were quantified in duplicate with a four-plex bead-based assay (Bio-Plex Pro™ Human Cytokine, Bio-Rad, Hercules, CA, USA) on a Bio-Plex 200; assays with R^2^ < 0.99 or CV > 15% were repeated. Detection limits were as follows: IFN-γ 0.12, IL-1α 0.08, IL-1β 0.10, TNF-α 0.09 pg mL^−1^. Full blood counts were generated on a Sysmex XN-1000 analyser (daily e-Check XR calibration). The mycobacteriology laboratory processed all respiratory specimens (cases and controls) with Auramine–O smear microscopy, MGIT 960 culture, Löwenstein–Jensen subculture, and conducted proportional drug-susceptibility testing per WHO technical guidance.

### 2.5. Statistical Analysis

Analyses were performed in Python 3.9 using SciPy 1.13, StatsModels 0.15, and Pingouin 0.5.4. Shapiro–Wilk tests and kernel-density plots were used to assess normality. Continuous variables were mean ± SD (Gaussian) or median (IQR) (non-Gaussian); Welch’s *t*-test or Mann–Whitney U were used to compare groups. Levene’s test was used to verify homoscedasticity. Categorical variables were n (%), compared with Pearson’s χ^2^ (Yates’ correction) or Fisher’s exact test. Spearman’s ρ was used to examin cytokine and NLR interrelations; Bonferroni correction adjusted α to 0.005 for 10 pairwise tests.

In-hospital mortality predictors were subjected to univariate logistic regression; covariates with *p* < 0.15 were retained in multivariable models (backward stepwise, exit *p* = 0.10). Model performance was evaluated with the Hosmer–Lemeshow χ^2^ and AUROC. LOS was modelled via quasi-Poisson regression to address over-dispersion; effects are incidence-rate ratios (IRR) with 95% CI. Sensitivity analyses (i) excluded subjects with incomplete cytokine panels and (ii) utilised multiple imputation by chained equations (m = 5, predictive mean matching). All tests were two-tailed; *p* < 0.05 denotes statistical significance unless Bonferroni-adjusted. The attained sample affords 92% power (α = 0.05) to detect a 5 pg mL^−1^ TNF-α difference and 88% power to detect a 10% relative change in LOS (SD 6 days).

## 3. Results

### Patient Demographics

The TB cohort was characterized by a distinctly male predominance, reflecting occupational and behavioural exposure trends that have been documented nationally; nearly four in five patients were men compared with just over one-third of community donors, which yielded a highly significant χ^2^ statistic (χ^2^ = 18.17, *p* < 0.001). Although the mean age difference of 3.5 years did not reach conventional significance (*p* = 0.096), the wider standard deviation among cases (14.8 years) underscores the age-heterogeneous nature of Romanian TB. The mean case BMI was 22.4 kg·m^−2^ (Table 1).

The cohort exhibited heavy exposure to conventional TB risk factors: 76.6% were current smokers and 36.4% reported alcohol consumption (29.9% chronic, 6.5% occasional). Known household contact with TB was documented in 16.9% of the patients, and nearly one-third (31.2%) had experienced at least one episode of COVID-19. Fewer than half (45.5%) were employed at the time of diagnosis. Symptomatically, 96.3% presented with active complaints, most commonly of <4 weeks’ duration (41.3%); a further 54.9% reported symptoms persisting beyond one month. The patients’ nutritional status was skewed towards normal weight (58.7%), but 27.5% were underweight and 10.0% were overweight. Comorbid conditions were common—31.2% had ≥1 chronic illness—and the median hospital stay was 29 days (IQR 23–34), as seen in Table 2.

**Figure 1:**
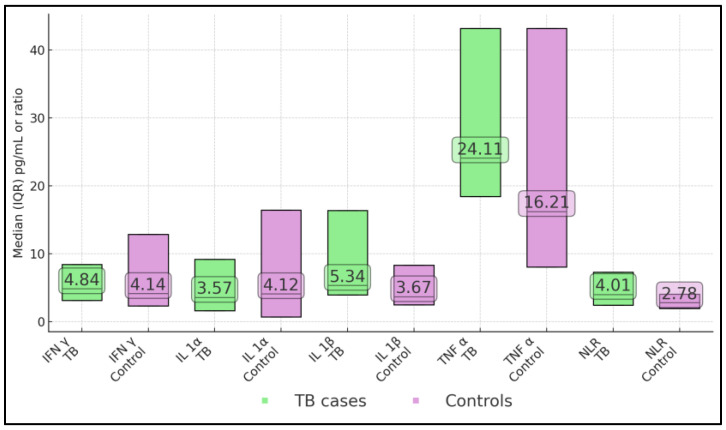
Circulating cytokines and NLR in TB cases versus controls.

Table 3 delineates the inflammatory milieu differentiating active TB from ostensibly healthy subjects. Contrary to canonical paradigms, IL-1β showed a significantly higher level in the patients with TB (median 5.34 pg/mL) versus controls (3.67 pg/mL, *p* = 0.008). TNF-α was conspicuously higher in the patients with TB (median 24.1 pg/mL vs. 16.2 pg/mL, *p* = 0.009), which aligns with its established role in granulomatous inflammation and cachexia. IFN-γ and IL-1α showed no between-group differences, which highlights that single-time-point measurements may inadequately capture the dynamic range of these factors across disease stages. The neutrophil/lymphocyte ratio trended upward in TB (median 4.01) but did not reach significance (*p* = 0.071) (Figure 1).

Among the patients with TB, the four non-survivors displayed markedly higher systemic inflammation. The median TNF-α tripled relative to the survivors (71.43 vs. 23.20 pg/mL; *p* = 0.037), and the NLR more than doubled (7.84 vs. 3.70; *p* = 0.002). IL-1β showed a strong upward trend (18.07 vs. 3.56 pg/mL) without statistical significance (*p* = 0.079), whereas the differences in IFN-γ and IL-1α were non-significant (*p* > 0.20). These data indicate that extreme elevations of TNF-α and NLR co-occur with early mortality (Table 4). The borderline *p*-value, coupled with a two-fold NLR rise in non survivors, suggests biological relevance that may reach statistical significance in larger cohorts.

**Figure 2:**
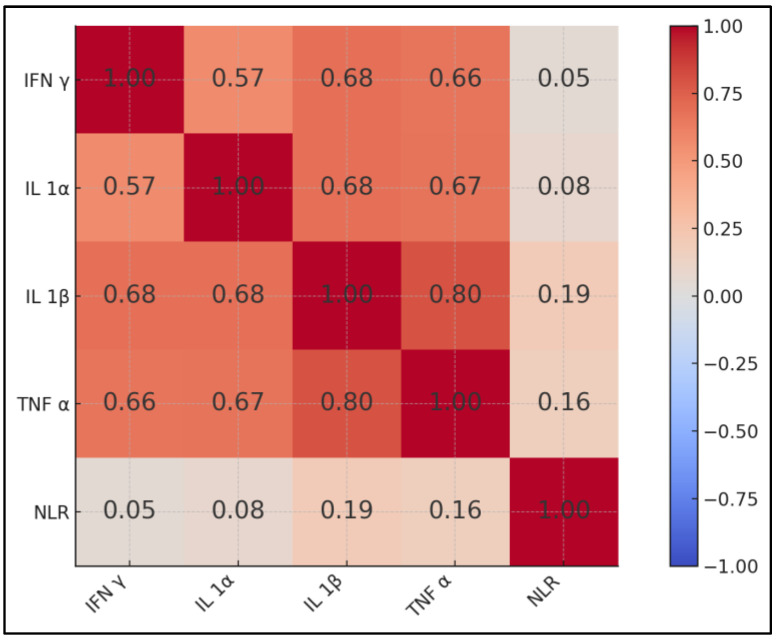
Correlation heatmap.

The cytokine network was densely interconnected: IL-1β correlated strongly with TNF-α (ρ = 0.80) and with both IFN-γ and IL-1α (ρ = 0.68 for each), all with *p* < 0.001. TNF-α also correlated robustly with IFN-γ and IL-1α (ρ ≈ 0.66–0.67). In contrast, the NLR showed only weak, non-significant associations with cytokines (ρ ≤ 0.19), which underscores its limited concordance with multiplex cytokine patterns captured here (Table 5). Although only four fatal events occurred, the odds ratio reflects the entire TNF-α range; higher ranking survivors contributed intermediate values that stabilised the regression slope. With all fatal-case TNF-α values being below 250 pg mL^−1^, the pattern represents exaggerated yet sub-storm systemic inflammation (Figure 2).

The median LOS did not differ by smoking status (28 vs. 31 days; *p* = 0.41) or comorbidity burden (28 vs. 29 days for ≥1 vs. none; *p* = 0.69). The nutritional status, however, stratified the stay duration: underweight patients had the shortest median LOS (27 days), normal-weight patients the longest (30 days), and overweight patients the briefest (23 days); overall variation across the BMI strata was significant (Kruskal–Wallis *p* = 0.03), which highlights BMI as the sole factor materially linked to hospitalization length in this cohort (Table 6).

## 4. Discussion

### 4.1. Analysis of Findings

The three-fold rise in the median TNF-α among our non-survivors (71 pg mL^−1^) corroborates the centrality of this cytokine in human TB pathogenesis. Mirzaei and Mahmoudi reported a similarly pronounced 1.7-fold serum excess in Iranian cases, proposing TNF-α as a readily deployable diagnostic adjunct [22]. Earlier broncho-alveolar studies from Taiwan demonstrated 30- to 40-fold elevations in alveolar fluid and macrophage transcripts, linking local over-production to parenchymal destruction [23]. Our data extend these observations by showing that systemic TNF-α independently forecasts in-hospital mortality after adjusting for age and bacillary load. Collectively, the regional concordance supports TNF-α-targeted host-directed therapy trials, but the narrow therapeutic window, highlighted by pre-clinical models that show bacterial over-growth when TNF-α is neutralised excessively, argues for adaptive dosing guided by early pharmacodynamic read-outs rather than fixed regimens.

Devalraju et al. demonstrated that TGF-β skews PBMC responses in HIV/TB co-infection, dampening IL-1β secretion and favouring mycobacterial persistence [24]. Conversely, Su et al. observed that patients whose chest radiographs improved after two months of therapy showed the coordinated down-regulation of TNF-α and IL-1β and reciprocal up-regulation of IL-10 [25]. Our donors may thus represent a “trained-immunity” baseline, whereas cases sampled weeks into illness exhibit inflammasome exhaustion. 

The very strong IL-1β–TNF-α correlation (ρ = 0.80) in our matrix supports a coordinated regulatory loop that has mechanistic precedent. Harris et al. showed that IL-1β synergises with IFN-γ to amplify p38-driven MMP-9 release, accelerating blood–brain barrier degradation in the central-nervous-system TB [26]. The same synergy likely underlies pulmonary cavitation and raises caution about using single-cytokine antagonists; the selective dampening of TNF-α could unmask IL-1β-mediated metalloproteinase activity. Our finding that the NLR is poorly aligned with the cytokine cluster reinforces the finding that inexpensive haematologic surrogates cannot fully substitute for multiplex assays when probing such interdependent networks.

Although the median NLR nearly doubled in fatal cases (7.8 vs. 3.6), the biomarker showed weak overall correlation with cytokines, echoing heterogeneous studies in the literature. Han et al. identified an NLR > 16 as an independent predictor of acute respiratory-distress development in miliary TB [27], whereas a 1,233-patient Chinese study found that the NLR was informative for cavitary disease but inferior to CRP-derived indices in multivariate models [28]. Our intermediate cut-offs (≥7) suggest that NLR’s prognostic bandwidth shrinks once hospital admission selects for moderate-to-severe cases. Practically, this means that the NLR should be interpreted against disease-stage-specific reference ranges and, ideally, combined with cytokine or transcriptomic read-outs to avoid false reassurance in patients whose neutrophil compartment is suppressed by sepsis or chemotherapy.

Participants who were underweight stayed a median three days longer than their overweight peers despite comparable bacillary loads—an observation substantiated by the Chinese NRS-2002 study, where patients with nutritional risk experienced significantly prolonged hospitalisation and higher complication rates [29]. A recent Brazilian survey adds population-level context: 26% of patients with ambulatory TB were underweight and 64% reported moderate-to-severe food insecurity, with their BMI, vitamin D status, and educational attainment jointly predicting their treatment adherence [30]. These data argue for integrating early anthropometry and micronutrient assessment into TB admission pathways in Romania. Nevertheless, interpretation of this study’s results should account for multiple comorbid conditions and patient risk factors that can alter these findings [31,32,33,34,35]. Simple interventions—energy-dense supplements, vitamin-D repletion, and social-assistance referrals—may yield disproportionate gains in patients’ LOS and mortality, particularly when combined with inflammatory profiling to target adjunctive anti-cytokine therapy for those at the highest biological risk. Future studies should incorporate IL-10, a known counter regulator of TNF-α, to determine whether the IL-10/TNF-α ratio further refines risk stratification.

### 4.2. Study Limitations

Several caveats temper the interpretability of our findings. First, the retrospective, single-centre design precludes causal inference and limits the external validity to similar tertiary settings in Eastern Europe. Although we enrolled consecutive culture-confirmed cases, the 80-patient sample affords modest power—particularly for mortality analyses in which only four events occurred—raising the possibility of type II error for secondary outcomes such as the NLR and cytokine interactions. Biomarker measurements were obtained at a single time-point without longitudinal follow-up, which prevented assessment of dynamic trends that could clarify the temporal relationship between cytokine modulation and the clinical course. Additionally, HIV testing was not uniformly documented, nutritional markers beyond BMI were unavailable, and no adjustment was made for the lineage or drug-resistance profile of Mycobacterium tuberculosis, all of which can influence host inflammatory signatures and outcomes. Also, we could not compare TNF-α/IL-1β with acute phase proteins such as CRP or ADA because the project funding did not cover these assays. Because our hospital is a tertiary referral centre, selection bias towards complicated cases is possible. We did not stratify the outcomes by radiological severity scores, which may have obscured lung burden effects on cytokine release. 

## 5. Conclusions

In this Eastern-Romanian cohort, pulmonary tuberculosis exhibited a distinct hyper-TNF-α systemic profile that strongly correlated with early in-hospital mortality, while the levels of inflammatory cytokines such as IL-1β were significantly higher than in controls. Traditional behavioural risk factors—smoking, alcohol use, and comorbidity burden—did not predict the length of stay or death once patients were hospitalized; instead, under-nutrition emerged as the principal modifiable determinant of prolonged admission. The observed TNF-α and IL-1β levels, coupled with the weak concordance between cytokine concentrations and the neutrophil/lymphocyte ratio, suggest that multiplex cytokine assessment offers superior prognostic granularity over routine haematologic surrogates. Integrating rapid TNF-α testing and systematic nutritional evaluation at admission could enable early risk stratification, targeted host-directed therapy trials, and resource optimization. Nevertheless, these preliminary findings warrant prospective, multi-centre validation before their integration into national TB triage algorithms.

## Figures and Tables

**Table 1:** Baseline demographic comparison between TB patients and controls.

Variable	TB (n = 80)	Controls (n = 40)	*p*-Value
Age, years (mean ± SD)	50.1 ± 14.8	46.6 ± 8.4	0.096
Male sex, n (%)	63 (78.8)	15 (37.5)	<0.001

Abbreviations: TB = tuberculosis; SD = standard deviation. Notes: Age is expressed as mean ± SD and was compared with Welch’s *t*-test; sex proportions were compared with Pearson’s χ^2^ test (Yates’ correction). *p*-values are two-tailed.

**Table 2:** Baseline behavioural and clinical characteristics of pulmonary tuberculosis cases (n = 80). Values are n (%) unless otherwise indicated.

Variable	n (%)
Current smoker	59 (76.6)
Chronic alcohol use	23 (29.9)
Occasional alcohol use	5 (6.5)
Known household TB contact	13 (16.9)
≥1 prior COVID-19 episode	24 (31.2)
Currently employed	35 (45.5)
Symptomatic at presentation	77 (96.3)
Symptom duration	
<4 weeks	33 (41.3)
4–12 weeks	23 (28.7)
>12 weeks	21 (26.2)
BMI category	
Underweight (<18.5 kg·m^−2^)	22 (27.5)
Normal (18.5–24.9)	47 (58.7)
Overweight (≥25)	11 (13.8)
≥1 comorbidity	25 (31.2)
Single comorbidity	17 (21.2)
≥2 comorbidities	8 (10.0)
Median length of stay, days (IQR)	29 (23–34)

Abbreviations: BMI = body mass index; COVID-19 = coronavirus disease 2019; IQR = inter-quartile range; LOS = length of stay. Notes: Values are n (%) unless otherwise stated. LOS is reported as median (IQR).

**Table 3:** Circulating cytokines and NLR in TB cases versus controls.

Marker	TB Median (IQR) pg/mL	Control Median (IQR) pg/mL	*p*
IFN-γ	4.84 (3.11–8.41)	4.14 (2.31–12.87)	0.527
IL-1α	3.57 (1.62–9.19)	4.12 (0.68–16.40)	0.599
IL-1β	5.34 (3.93–16.35)	3.67 (2.48–8.31)	0.008
TNF-α	24.11 (18.42–43.15)	16.21 (8.02–43.15)	0.009
NLR	4.01 (2.44–7.28)	2.78 (1.93–4.05)	0.071

Abbreviations: IFN-γ = interferon-gamma; IL-1α = interleukin-1-alpha; IL-1β = interleukin-1-beta; TNF-α = tumour-necrosis-factor-alpha; NLR = neutrophil/lymphocyte ratio; IQR = inter-quartile range. Notes: Data are median (IQR). Between-group differences were assessed with the two-tailed Mann–Whitney U test. Lower limits of detection for the multiplex assay were IFN-γ 0.12 pg mL^−1^, IL-1α 0.08 pg mL^−1^, IL-1β 0.10 pg mL^−1^, and TNF-α 0.09 pg mL^−1^.

**Table 4:** Cytokine concentrations stratified by in-hospital survival in TB patients.

Marker	Survivors (n = 76) Median (IQR)	Non-Survivors (n = 4) Median (IQR)	*p*
IFN-γ	4.84 (3.02–8.32)	8.66 (4.67–19.26)	0.22
IL-1α	3.57 (1.62–8.37)	9.09 (1.89–26.41)	0.74
IL-1β	3.56 (2.43–8.13)	18.07 (4.94–46.03)	0.079
TNF-α	23.20 (18.19–37.54)	71.43 (48.69–140.22)	0.037
NLR	3.70 (1.95–5.55)	7.84 (2.55–9.41)	0.002

L-1β = interleukin-1-beta; TNF-α = tumour-necrosis-factor-alpha; NLR = neutrophil/lymphocyte ratio; IQR = inter-quartile range. Notes: survivors (n = 76) and non-survivors (n = 4) were compared using the two-tailed Mann–Whitney U test because cytokine distributions were non-Gaussian.

**Table 5:** Spearman correlation matrix of inflammatory markers and NLR.

Variables	IFN-γ	IL-1α	IL-1β	TNF-α	NLR
IFN-γ	1	ρ = 0.57 *	0.68 *	0.66 *	0.05
IL-1α	0.57 *	1	0.68 *	0.67 *	0.08
IL-1β	0.68 *	0.68 *	1	0.80 *	0.19
TNF-α	0.66 *	0.67 *	0.80 *	1	0.16
NLR	0.05	0.08	0.19	0.16	1

Abbreviations: IFN-γ = interferon-gamma; IL-1α = interleukin-1-alpha; IL-1β = interleukin-1-beta; TNF-α = tumour-necrosis-factor-alpha; NLR = neutrophil-to-lymphocyte ratio. Notes: Entries are Spearman rank-correlation coefficients (ρ). Asterisks indicate *p* < 0.001 after Bonferroni correction for 10 pairwise tests.

**Table 6:** Length of stay (LOS) subgroup analysis in TB cohort.

Subgroup	n	Median LOS (Days)	Comparator n	Median LOS	*p*
Smoking vs. non-smoking	59	28	18	31	0.41
≥1 comorbidity vs. none	25	28	52	29	0.69
BMI categories				0.03 †	
Underweight	22	27	—	—	
Normal	47	30	—	—	
Overweight	8	23	—	—	

Abbreviations: LOS = length of stay; BMI = body mass index. Notes: LOS values are medians. Smoking and comorbidity subgroups were compared with the two-tailed Mann–Whitney U test; BMI categories were compared with the Kruskal–Wallis test († symbol in table denotes this omnibus comparison).

## Data Availability

The data presented in this study are available on request from the corresponding author.

## Abbreviations

The following abbreviations are used in this manuscript:

ADA	Adenosinedeaminase
ACE	Angiotensin-convertingenzyme
AUROC	Area under the receiver-operating-characteristic curve
BCG	Bacille Calmette–Guérin(vaccine)
BMI	Body mass index
COPD	Chronicobstructive pulmonary disease
COVID-19	Coronavirus disease 2019
CRP	C-reactive protein
CT	Computed tomography
CXCL	C-X-C motif chemokine ligand
ECDC	European Centres for Disease Prevention and Control
EPTB	Extrapulmonary tuberculosis
EU	European Union
GBD	Global Burden of Disease study
HDT	Host-directed therapy
HIV	Human immunodeficiency virus
ICH	International Conference on Harmonisation
IFN-γ	Interferon-gamma
IL-1α	Interleukin-1-alpha
IL-1β	Interleukin-1-beta
IL-6	Interleukin-6
IL-10	Interleukin-10
IRB	Institutional review board
IRR	Incidence-rate ratio
IQR	Inter-quartile range
LOS	Length of stay
MDR-TB	Multidrug-resistant tuberculosis
MGIT	Mycobacteria Growth Indicator Tube (culture system)
miR	Micro-RNA
MMP-9	Matrixmetalloproteinase-9
NLR	Neutrophil-to-lymphocyte rratio
NTP	(Romanian) National Tuberculosis Control Programme
OR	Odds ratio
PBMC	Peripheral-blood mononuclear cell
pSWE	Point shear-wave elastography
ROC	Receiver-operating-characteristic curve
R^2^	Coefficient of determination
SD	Standard deviation
STROBE	Strengthening the Reporting of Observational Studies in Epidemiology
TB	Tuberculosis
Th1	Type-1 helper T-cell response
TNF-α	Tumour-necrosis-factor-alpha
WHO	World Health Organization
